# Ten simple rules for building and maintaining a responsible data science workflow

**DOI:** 10.1371/journal.pcbi.1012232

**Published:** 2024-07-18

**Authors:** Sara Stoudt, Yacine Jernite, Brandeis Marshall, Ben Marwick, Malvika Sharan, Kirstie Whitaker, Valentin Danchev

**Affiliations:** 1 Department of Mathematics, Bucknell University, Lewisburg, Pennsylvania, United States of America; 2 Hugging Face, Inc., New York, New York, United States of America; 3 DataedX Group, Atlanta, Georgia, United States of America; 4 Department of Anthropology, University of Washington, Seattle, Washington, United States of America; 5 The Alan Turing Institute, London, United Kingdom; 6 School of Business and Management, Queen Mary University of London, London, United Kingdom; Dassault Systemes BIOVIA, UNITED STATES OF AMERICA

Contributors and beneficiaries of data-intensive research have become increasingly concerned about social and ethical risks from data science and machine learning applications [1–6]. Instances of unethical use of technology and harms caused to vulnerable communities have made it even more urgent for researchers to broaden the considerations of ethics and societal impact in their research. There has been a proliferation of ethical guidelines [7–10], checklists for responsible research [11,12], and teaching materials [13] encouraging the application of good research practices in all areas of data science research, including machine learning (ML), artificial intelligence (AI), and natural language processing (NLP). While encouraging, there is also a risk that ethical considerations from guidelines and checklists may be added to a project as an afterthought unless such considerations are incorporated into the research process from the onset so that data science can be performed responsibly by design (in a similar vein as advocated for by Open Science by Design [14]). To help enable this goal of incorporating ethics through the entire research process, we outline 10 simple rules of a responsible data science workflow.

A responsible data science workflow scaffolds practices and processes of ethical research, defined by the European Commission as “an approach that anticipates and assesses potential implications and societal expectations with regard to research and innovation, with the aim to foster the design of inclusive and sustainable research and innovation” [15]. We stress that this approach should be considered at each stage of the data science lifecycle [6,16,17]—ranging from team assembling and research design to data collection and evaluation, model building, model evaluation, and reporting. Data science projects often involve multiple teams and contributor groups, and hence, it is our ethical responsibility to embed practices for inclusive and collaborative research as well. A responsible data science workflow identifies and invites different stakeholders, possibly with different interests, expertise and access to resources [18], to participate in the workflow and provide feedback, especially those who are affected by data science research, including research subjects, collaborators, community members, and those from marginalized groups (see Fig 1).

**Fig 1:**
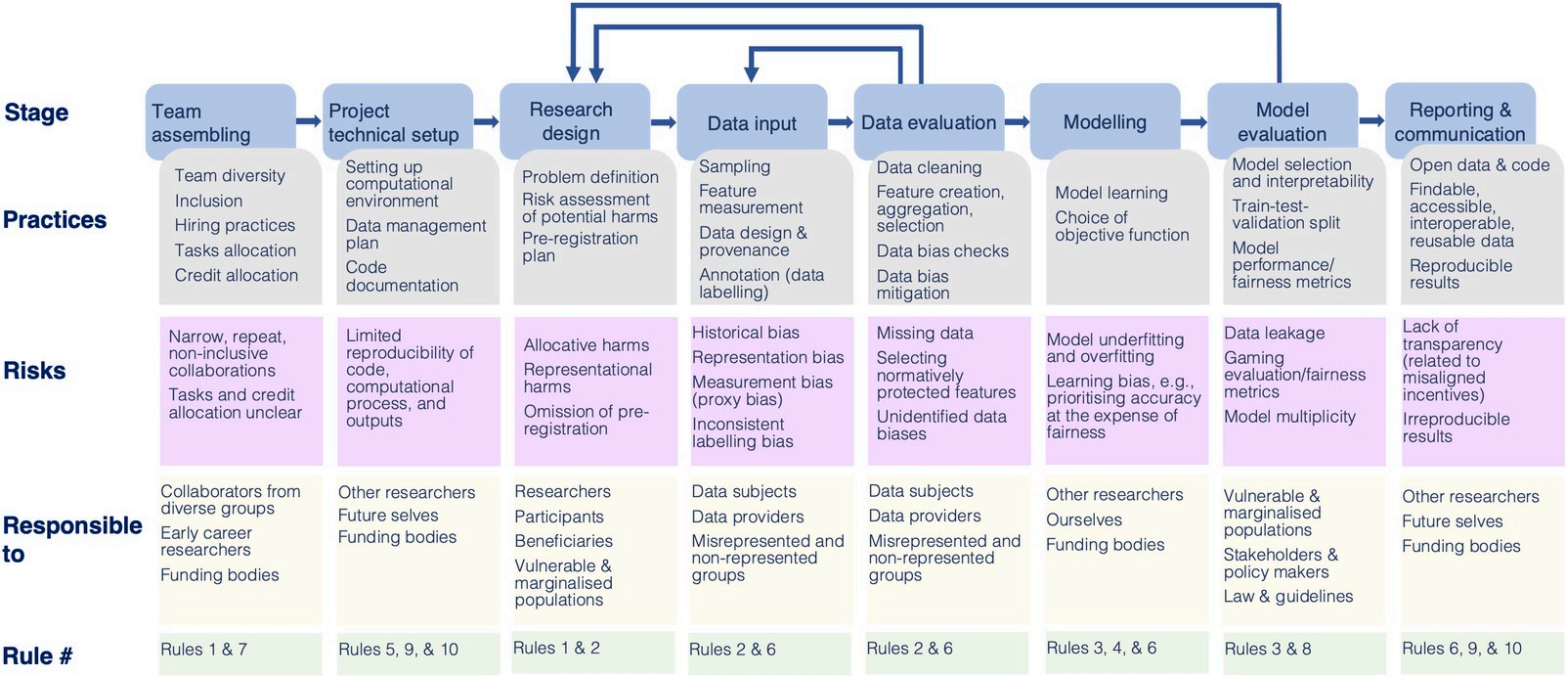
Components in a responsible data science workflow. For each stage in the data science life cycle, we identify associated practices of research ethics, risks, and responsibilities towards key stakeholders. This figure is inspired by [6,16].

Historically, questions and considerations around research ethics have primarily focused on the issues of privacy, confidentiality, and rights of research participants (or data subjects) [19]. More recently, attention has also been placed on fairness and bias of prediction models and modelers’ responsibility towards users and members from minorities and underrepresented groups as well as regulations concerning data use and privacy as well as explainability of outputs to those affected [4]. The movement towards openness and research transparency have emphasized the relevance to the current scientific ethos of the Mertonian norm of “communality”—that scientific objects such as data, methods, and findings are common property to the scientific community and that advancements in science depends on open communication and sharing [20–24].

Responsibilities are myriad, but there are a few key ones that span a data-intensive project. Maintaining transparency of research objects through documentation for fellow researchers and funding bodies means that the plan for the analysis is written down, shared, and followed, and the approach used to collect, clean, and preprocess data can be followed by someone outside of the research team [20]. Working towards model interpretability is also an important responsibility, related to transparency, because those affected by the model or governing bodies who rely on the model need to have a full understanding to make informed decisions. There is a responsibility to ensure diversity, inclusion, and fair recognition of all contributors and collaborators on a project such that team members contribute a variety of experiences to the project, feel welcome and supported by their teammates, and get recognition for their contributions when results are shared. A responsible data science workflow should embed ethical and social considerations across the data science lifecycle and across the practice of collaborative research, while acknowledging that one or another consideration may be more pertinent to a particular setting.

Research workflows need not to be narrowly centered on the process of data analysis and software tool-building alone. They can and should integrate data analysis holistically with broader considerations of good collaborative and computational practices. These practices include the use of FAIR (Findable, Accessible, Interoperable and Reusable) research objects including data and tools, supported by initiatives like the European Open Science Cloud [25] that aim to streamline data sharing for re-use of data, Free and Open-Source Software (FOSS), inclusive approaches, clear communication, and an attention to ethics and the social impact of the research (as broadly defined in *The Turing Way*) [15,26,27]. In the context of Indigenous data, the CARE (Collective benefit, Authority to control, Responsibility, and Ethics) principles should be applied to complement FAIR practices ensuring the “use of data aligns with Indigenous rights, is as open as determined by Indigenous communities, is purposeful, and enhances the wellbeing of Indigenous Peoples” [28]. Reproducibility is certainly something to aspire to, but a workflow can push further towards responsibility by considering both the technical and social aspects of the project.

Further, responsible research workflows not only apply reproducible practices while engaging with ethical approaches throughout the whole process but can also adapt as the need emerges. Many discussions about reproducible and responsible workflows are about how to set up the “right” workflow, while acknowledging that the “right” workflow today might not be “right” tomorrow. Throughout the rules presented, we discuss how researchers can navigate the process of changing research workflows given new contexts and constraints.

In these 10 simple rules for building and maintaining responsible data science workflows, we walk through the lifecycle of a project and consider how a research team can responsibly manage both the technical and social aspects of the project, adapting when necessary. These 10 rules are by no means prescriptive as we recognize the complexities surrounding responsible research and the heterogeneity of data science applications across research communities and fields. In addition, the iterative nature of exploration and refinement within a project can lead to nonlinearity in the workflow that can make data and computationally intensive research challenging. Nevertheless, we hope the rules can help interested researchers build and maintain a responsible workflow and collaborations.

## Rule 1: Explicitly consider ways in which your research findings could be used to do harm

The beginning of a research project is often full of energy and promise. At this stage, it can be hard to properly assess the ethical implications of a research project before a team has collaboratively set the overarching goals and decided on its next steps. Issues like the input data not being as representative as a team initially thought, or others, like overgeneralizing the findings such that they make inadvisable recommendations to a vulnerable population, could start to appear. Therefore, it is important to embed checkpoints in the early planning stage for the research teams to seriously reflect on the unintended consequences of their work.

Early reflection can happen while the research team conducts a literature review as part of their preliminary work to learn about the current state of the art and consider how to place their new idea. As the team reads about other projects that have approached a similar problem to the one they are interested in solving, they could be prompted to categorize past projects in terms of types of negative impacts they have the potential to impose. For example, are there any privacy concerns that arise from an effort to make input data openly available, or is there performance bias of a predictive algorithm applied to human decisions that could lead to unfair outcomes for different people?

Beyond the academic literature, what disaster stories have been heard related to the type of data or approach the team is considering, perhaps in the news or collected in books like *Algorithms of Oppression*: *How Search Engines Reinforce Racism* [29], *Race After Technology* [30], and *Weapons of Math Destruction* [4]? Could the described incidents reappear in the proposed project? Research teams can even learn from the entertainment that its members consume. What dystopian future could result from the work? Experts in data-related and technology fields have even started to bridge the gap between traditional dystopian worlds and specialized scenarios that are informed by the work they do (e.g., [30–32]).

As Skirpan and Yeh warn: “with the blinding light of promise glistening, we must be careful not to miss that there are consequences and dangers” [33]. They advocate for a speculative analysis of the field, mixing ideas from formal risk analysis with those of speculative fiction. Similarly, Gaskins advocates for taking inspiration from Afrofuturism creatives and speculative designers to question algorithms [34]. If the algorithm is designed for use by an “average” user, how do atypical users fare? Are predictive algorithms just as accurate for data points representing all demographics? This idea of constant questioning, even from the beginning, is emphasized in Marshall’s book, *Data Conscience*: *Algorithmic Siege on our Humanity*, which connects the principles of data, technology, and human ethics and outlines key motivating questions to consider [2].

Disasters aren’t the only thing to think about; seemingly innocuous decisions can have biases baked in and lead to unintended consequences. For example, suppose you are in charge of collecting data to inform a policy change about the maximum building height allowed in a neighborhood. You may look at the heights of buildings that are listed in prior permits over time, keep track of how limits in the legislation have changed, and release a survey about preferences for people who live in the neighborhood. So far, this scenario might seem pretty straightforward and low risk for ethical complications.

However, let’s dig in a bit more. What about the people who cannot afford to live in the neighborhood but commute there for work? The commute may take up considerable free time and so they like to take advantage of the green space nearby their office building to eat their lunch and get some fresh air. Higher buildings might block the sun and make that space inhospitable for plants, wildlife, and lunch eaters alike. You won’t know about these people’s preferences though because you only surveyed people who live in the area.

Let’s also consider who the policy makers have been in this area. Are their demographics and stances reflective of the population? Who has been pushed out of this neighborhood by previous changes in policy, and how might that affect what you see in the building height data? By making your decision solely based on information that you have access to in the historical record, you may be perpetuating historical biases.

Going through expansive reading, reflection, and questioning process, in scenarios big and small, not only helps avoid unintended consequences in the future but can also make the intended audience or user base that the team is responsible to more concrete early on.

## Rule 2: Question your inputs: What is the data provenance and what privacy concerns or biases might be at play?

Questioning your data inputs—how data was designed and for what purposes and uses—should be an integral part of a responsible data science workflow. Data science research has historically prioritized model performance but many recent concerns about bias and fairness could be traced back not only to the models but also to the data used to train the models [5,6,35,36]. Because data is central for the development, evaluation, and validation of data science and ML models, the impact of deviations from originally intended use and data quality on model outputs may be considerable.

Data science research operates in a specific data governance regime of how data is generated, collected, and shared. Data science research typically uses “readymade” data [19] which was designed, generated, and collected by governments, public sector organizations, and companies for purposes that were different from the specific research question being pursued. In contrast to “custom made” data, where researchers have clearly stated intentions and control in the process of data production, many properties of readymade data may be unspecified or unclear for the researchers using the data, introducing possible social and ethical harms from models trained on the data. Both types of datasets require further scrutiny when used as data inputs. It is important that open data frameworks along with FAIR and CARE principles are applied to enable purposeful use and reuse of data that promote equitable access and sharing of benefits [28].

When you examine potential data for your research, you may first evaluate for data transparency and provenance: Who funded and collected the data, how is the data distributed, and for what purposes and what intended uses? A useful starting point is provided in “Datasheets for Datasets” [35] that outlines a standardized template for documenting data, including motivation, composition, collection process, recommended uses, distribution, and maintenance of the data. Datasheets for Datasets can facilitate responsible (re)use of data. Questions like “For what purpose was the dataset created?”, “Was there a specific task in mind?”, and “If consent was obtained, were the consenting individuals provided with a mechanism to revoke their consent in the future or for certain uses?” highlight the need to carefully consider the implications and potential ethical quandaries that can arise when using data in a new context. If you are planning to use a dataset, a good starting point will be to check for an available Datasheet documenting data provenance, characteristics, and potential risks. If a dataset does not have a datasheet, as would be the case with many datasets, especially earlier ones, a possible solution would be to contact data creators regarding data characteristics. Data hubs can also provide an entry point to search datasets and to standardized data documentation. Marshall’s book also provides a list of questions to go through during the data sourcing process to help navigate the use of preexisting data, the need to collect data, the rights of use, and the logistical structures of use (Table 5.1 in [2]).

Dataset documentation may not anticipate all potential biases in your particular application. An important part of your responsible workflow is to evaluate for potential data-associated biases [5,6]. This includes representation or sampling bias arising when the data used to train a data science model underrepresents some parts of the population. As a result, the trained model may fail to generalize for an underrepresented population. A related, historical bias, occurs when, even in the absence of sampling bias, some population groups are underrepresented in the data due to structural disparities or inequalities in the past. A third family of biases refers to measurement bias that arises due to inaccuracies in how variables, features, or labels are measured or classified. In big data research, measurement bias often occurs when readymade measures are used for proxies of unavailable true values. For example, a model that uses health costs as a proxy for health needs was found to discriminate against black patients [3]. Because black patients incur lower health care costs due to unequal access to treatment, for patients with otherwise the same levels of health needs, the algorithm would falsely conclude that black patients are healthier than white patients, thereby prioritizing white patients for treatment while underestimating the health needs of black patients [3].

Another ethical consideration is not a bias per-se but has to do with informed consent. Participants may have consented to have their data used to answer a particular research question, but may not feel comfortable extending that consent to future research questions. Just because data exists, doesn’t mean it should be used. For example, patients in a new drug trial may consent to having their data be shared for other future medical studies but may not want their data passed along to insurance companies for risk analysis studies.

Data biases should be identified and mitigated at the early stages of the data science lifecycle. However, this is often not the case. A recent survey of the ML literature indicates that mitigation efforts are overwhelmingly focused on the modeling stage, even though problems and biases are often identified and measured at earlier stages of problem formulation and data collection and processing [16].

There is no “one size fits all” data bias mitigation strategy. The effectiveness of data bias mitigation strategies would depend on the sources of bias and on the application [6]. For example, in the presence of representation bias, data augmentation through the collection of additional data samples of underrepresented groups could be an effective bias mitigation strategy. However, in the presence of historical biases, the collection of additional data would be an insufficient mitigation strategy. To mitigate historical biases, systematically under- or oversampling may be part of a solution but you would typically also need fairness approaches (see Rule 3) that can deal with biases in observational data. One such approach, informed by the literature on causal inference [37], is counterfactual fairness, which considers a model outcome to be fair to an individual if it is the same in reality as it would be in a counterfactual world in which the individual is part of a different sociodemographic group [38,39]. For example, for the task of predicting success in law schools, a model would be counterfactually fair if the predictions for applicants with observed race and sex are comparable to the predictions given applicants’ counterfactual race and sex categories [39].

However you decide to assess bias in your input data, including the outputs of your bias checks as well as bias mitigation strategies in the materials you share can help others learn from your approach, and the transparency can help build trust within communities you are responsible to.

## Rule 3: Evaluate progress with respect to goals and with a process for detecting bias, unfairness, and gaming of metrics

There have been concerns about the bias and fairness of data science and machine applications in high-stake domains ranging from healthcare to the justice system [1,3]. Business or research goals may be in conflict with bias and fairness goals, so it is important to consider them together rather than one at a time. For example, Marshall discusses a hiring tool built by Amazon in the mid 2010s (Chapter 3 in [2]). The tool was motivated by the vast scale of resumés coming in and the business need to sift through them more efficiently. However, unintended consequences were revealed when the tool downweighted resumés of those from minority groups—diversity was not explicitly a goal of the algorithm.

Many metrics have been proposed to evaluate fairness and bias in data science models [38]. Yet, in comparison to the set of metrics used to evaluate the performance of ML models, there is no agreed-upon set of metrics used to evaluate for fairness that researchers can use. This is understandable given that fairness criteria have been found to be incompatible and no method or metric can satisfy desirable fairness criteria simultaneously [40–42]. The incompatibility between fairness criteria implies that fairness metrics cannot be easily plugged into a model pipeline. Instead, researchers need to check for bias and fairness in their models in the context of the communities they are responsible to, their policy goals and social values, and the ethics relevant to the application in question. If you evaluate biases in your research design and data, the next step is to evaluate and potentially fix your model.

In recognition of the limitations of any particular fairness metric, frameworks have been recently developed to audit models and potentially mitigate social and ethical biases through model transparency, interpretability, explainability, and fairness. Open-source tools for detecting biases and unfairness have been proposed, including Aequitas [43], Fairlearn [44], and AI Fairness 360 [45]. A particular advantage of such tools is that instead of focusing on a particular stage of the ML process or a particular bias, they allow a systematic examination of models for various biases throughout the model development and application lifecycle. However, you need not limit your responsible workflow to such tools. In many contexts, tools and metrics may not be the appropriate approach to address model interpretability and fairness. More effective solutions could be found, for example, in the way organizations and teams are formed. Involving diverse people in problem definition, data collection, and model evaluation may provide a more sustainable solution.

The flexibility of such tools poses some risks as well. There are a plethora of methods and measures for model debiasing and fairness. While such diversity is helpful, the very choice of fairness criteria and potential trade-offs between fairness and accuracy as well as numerous fairness metrics increase researcher degrees of freedom and flexibility. As a consequence, unless preregistered in consultation with stakeholders, the application of an arbitrary fairness metric may ensure neither fairness nor reproducibility but rather opportunities for gaming the fairness metrics (see Goodhart’s law) [46]. Issues of fairness evaluations can be exacerbated in the context of related reproducibility issues in data and ML-based sciences such as model multiplicity (when for the same prediction task, there are multiple models that have equal accuracy but differ in their individual predictions and fairness properties) [47] and data leakage (when information related to the target variable in the test data is “leaked” to the training data) [48]. This emphasizes the importance of good research and software practices that we discuss in Rule 6 below. The same caution applies to model interpretability and explainability. One would need to ask the question: Interpretable to whom? Researchers, policy-makers, or end users? In some settings, current hopes for interpretable and explainable AI may be unrealistic, and a rigorous internal and external validation of models may better achieve the goals of interpretability and fairness in a responsible data science workflow [49,50]. All of these considerations highlight the fact that model evaluation and bias mitigation cannot be automated in a pipeline but require continuous integration of policy and social goals, domain knowledge, and model specifications.

## Rule 4: Embrace iteration: Goals and the metrics for measuring progress should be reevaluated and bias mitigation strategies improved as necessary

A lot can change in a data science project: the data can change (e.g., differential privacy approaches to data sharing are adopted by the United States Census), the context can change (e.g., new laws like the European Union’s General Data Protection Regulation impose new constraints), the impact can change (e.g., a tool built for one purpose like a “fun” face swap filter on Instagram, is used for something else like malicious deep fakes) [2,51,52]. If the input data experiences data shift, when the distribution of the data that a workflow is built with is different than the distribution of the data that the workflow is currently being used with, the validity of the outputs may be in jeopardy [53]. It may be necessary to not only change inputs, or an analysis approach, but also how outputs are evaluated to ensure continued accuracy and to mitigate differential impact of any future degradation. Beyond your assessment metrics, be open to this change more generally! Even welcome it by scheduling reflective assessments periodically. For ideas on what this process can look like, see Rule 8 which further discusses reflection.

Going a step further beyond bias mitigation strategies, ideas of algorithmic reparations remind us that sometimes the solution is not to debias but to rather use the bias to improve equity [54]. Davis and colleagues [54] use the Correctional Offender Management Profiling for Alternative Sanctions (COMPAS) tool as an example. COMPAS makes predictions about whether a defendant is likely to commit another crime, and this prediction is used to help make decisions about sentencing. A first metric of success may be to minimize false positives across the whole dataset. However, with an eye towards equity, the team might worry about the distribution of false positives across racial groups being uneven and update its metric of success to be equal false positive rates across groups. Going even further, even algorithms that result in “fair” errors, ones that are evenly distributed across demographics, may seem well intentioned, but this metric of success does not consider that the effect of an error may differ by demographics. As the team iterates on their goals, refining them based on their responsibility to those impacted by their decisions, they may consider that the impact of more severe sentencing may differ across racial groups and further refine their metrics of success. For example, Davis and colleagues [54] discuss the virtues of a reparative algorithm that would be proactive about this and “protect” groups that face “disproportionate risk.” One approach to algorithmic reparations is to work more closely with communities that you are responsible to so that the products are understandable and share a sense of trust through co-creation.

## Rule 5: Confirm the functionality and fairness of the overall workflow each time an element changes

Although a responsible workflow should be open to change by being iterative and adaptive, the logistics of navigating this change must also be considered. Best practices from software engineering, like those outlined in [55], can be adopted to make it easy to check that changes will not break other components of the project and help avoid pain points. If different members of a team are working on different components, this is especially important.

As different parts change, potentially at the same time, “gut checks” can help avoid disaster. Errors can occur in many types of project components, some involving the technical process like coding and data analysis errors and others involving the more informal workflow of collaborations including miscommunications between team members. For technical processes, “gut checks” could be formal unit tests [56,57] like those used in software engineering-style workflows or investigations of unexpected outcomes [58,59]; for non-technical processes, “gut checks” could be a conversation between all members of a team involved in the downstream processes of a proposed change before moving forward.

Once changes are ready to be formally included, the transition between the old and new versions should be organized such that if something goes wrong, there is a fail-safe, working approach that is defaulted to. A software engineering practice of continuous integration, where small changes are contributed frequently to a shared code base and run against tests to ensure continued functioning, can help avoid inducing errors and incompatibilities across multiple contributions to a system [60,61]. If a team anticipates making “breaking” changes, members need to consider the downstream effects of their user community, and act accordingly [62–64].

Consider a simple example where a project continually adds to a spreadsheet that it shares, along with the processing code, on GitHub. As the project starts out, the spreadsheet doesn’t have many rows, but as the project progresses, the file gets larger and larger. Eventually, it will hit the file size limit for a file (100 MiB) [65]. Having a back-up plan for data sharing when this happens and a process for navigating the transition in a transparent way without gaps in access to the data ensures continual functionality. Teams may do this by changing reference URLs to the data and ensuring that code further on in the pipeline references the most up-to-date storage location.

Often, as changes are being made, there should be a working prototype available in the meantime. However, there may be cases where service should be discontinued until changes are officially made. For example, if harm is currently being done, such as with a privacy breach or some other unintended consequence, having nothing might be better than having something, despite “losing face” in the short term (e.g., [66]). Building off of the earlier example, if each row is a de-identified record, data privacy may be upheld at the beginning of the project. However, as the records get continually updated, there may be more and more information tied to an anonymous user id that might make them more likely to be identifiable. Continually checking for adequate anonymity before updating the dataset publically is required to make sure the workflow does not break down in its promise to maintain participants’ privacy.

## Rule 6: Follow best practices for transparency, reproducibility, and documentation and follow FAIR principles

The first 5 rules have focused on the addition of a social responsibility layer to a technical workflow, but a responsible workflow also pairs a reproducible workflow with an ethical framework. Both computational transparency and reproducibility are critical for data-informed computational research [15,20,67–73]. Taking care with technical specifications can actually make the work more ethical in general, by increasing access and building trust through transparency and reliability.

At the phase when you design your research study, a good practice is to preregister your study. Preregistration is the practice of documenting your research plan (including research questions, hypotheses, and statistical analysis) and storing it in a public repository before observing your research outcomes [74,75]. By separating exploration from testing of predictions, preregistration brings researcher degrees of freedom to light [76] and helps protect researchers from biases that are otherwise hard to avoid [76,77], including possible selective reporting and overreporting of false positive results [74,75]. Preregistration strengthens model validation techniques such as train-test split and cross-validation which are widely used in data science and ML research to separate the phase of model exploration and fine-tuning from the testing phase as a way of avoiding overfitting [68]. Preregistration is also an opportunity to engage stakeholders early on in the project when the research team can still adapt the plan based on feedback before any formal testing takes place.

Computational reproducibility refers to the verification of results using “the same input data, computational steps, methods, and conditions of analysis” [71]. Transparency of computational workflow, code, data, and materials documenting the research process enables reproducibility but also has an added layer of accountability to the groups that the project is responsible to. Then, at any point in the research process, those impacted by the work can check in and see how the project is going, weigh in on decisions, and give feedback on next steps.

At the stage of research analysis, you can improve research transparency and reproducibility by avoiding or transitioning from point-and-click workflows and adopting coding scripts or computational notebooks such as Jupyter Notebook [78] and Quarto [79]. Computational notebooks are open-source web applications that allow you to create and share documents that contain code, equations, visualizations, and text. Most notebooks support various widely used open-source programming languages, including Python, R, and Julia. While a popular tool for data exploration [80], notebooks can also support your reproducible research workflow by integrating executable code, data inputs, results, and documentation within a single document [80–83]. Computational notebooks support reproducibility, but the tool itself is not sufficient for reproducible data analysis. You also need a reproducible research workflow [69,83], code documentation [84], and code review [85], all of which would help us transition from a “nonlinear, interactive, trial-and-error style of exploration to a more linear and reproducible analysis based on organized, packaged, and tested code” [86].

A data analysis may be reproducible but still contain errors or bugs that question the veracity of the research findings. One such issue is data leakage. In simple terms, data leakage arises when information related to the target variable in the test data is “leaked” to the training data. Kapoor and Narayanan [48] identified various sources of data leakage, including very simple errors like using the same dataset for both training and testing or incorrect preprocessing (e.g., the imputation of missing values is performed on the entire dataset instead of on the training data and the test data separately). As with computational reproducibility, to mitigate the risk of such errors and code, transparency of research code and data is essential.

At the phase of disseminating your research study, it is a good practice to make your research outputs—data, software, and associated metadata—“FAIR” such that they are more Findable, Accessible, Interoperable and Reusable [27,87]. This makes the work more inclusive by allowing anyone to discover your work, access its components, and reuse it to further their own research, not just those with particular resources at their disposal. When you are ready to share your project as a publication submission, preprint, or another public form of dissemination, you can use a guide [88] to make your dataset and its associated metadata available for others via archiving services such as Dryad, Zenodo, Open Science Framework (OSF), and Figshare. For private, sensitive, individual-participant data (IPD), you can use Trusted Research Environments (TRE) [89] and safe and secure storage platforms, such as Vivli for clinical trial data [90]. Many TRE platforms provide secure and transparent access to large healthcare and other potentially sensitive observational data, such as the National Health Service (NHS) Digital’s Trusted Research Environment [91] and OpenSAFELY [92].

You can also integrate your computational notebooks with version-control software such as Git, GitHub, and GitLab [72,83] so that others can access your code and its history, and possibly collaborate and/or reproduce your research. To enable others to execute your computational notebook interactively, you can package your notebook and underlying computational environment using various tools for containerization [93] and cloud-computing service (for example, Binder creates an executable Jupyter notebook in Julia, Python, or R). Making your computational workflow executable, interactive, and reproducible across platforms removes the need for an interested user to spend time and money downloading particular software and setting up their computing environment in a very specific way [78].

Tackling the full scope of reproducibility best practices is beyond the scope of this paper, but we point you to the many resources available to help you learn about best practices for reproducibility [15,20,72,81,82,94] and scientific computing [84]. For example, the Turing Way is an open, community-driven project “dedicated to making collaborative, reusable and transparent research ‘too easy not to do’” [15]. The R user community, including groups such as rOpenSci, have developed packages specifically to enable open and reproducible research, such as rrtools, workflowr, and usethis [82,95–97]. More recently, the BigScience Workshop [98] has brought researchers together to build and then evaluate large computational language models. You can also improve the transparency and reproducibility of your data science research by consulting reporting guidelines, such as the TRIPOD-AI (Transparent Reporting of a multivariable prediction model of Individual Prognosis Or Diagnosis-Artificial Intelligence), reproducibility checklists, such as “The Machine Learning Reproducibility Checklist” [99], “Model info sheets for detecting and preventing leakage” [48], and the “NLP Reproducibility Checklist,” and/or take part in the ML reproducibility challenges, such as the ML Reproducibility Challenge 2022 [100], aiming to reproduce work submitted to major machine learning conferences, including Neural Information Processing Systems (NeurIPS), International Conference on Machine Learning (ICML), International Conference on Learning Representations (ICLR), Annual Meeting of the Association for Computational Linguistics (ACL), and Association for Computing Machinery Conference on Fairness, Accountability, and Transparency (ACM FAccT).

## Rule 7: Apply fairness and inclusivity principles to your collaborators as well as your research topic

A responsible data science workflow practices what it preaches; turning inward when assessing use of inclusive practices as well as considering the outward impact of the work. Effective collaborations are as much about the social dynamics as they are about the technical aspects of the work. Fostering an inclusive environment for the entire team takes intentional effort across multiple stages of a research project from onboarding new teammates, to retaining them and providing support for growth [101].

Collaborative work requires division of labor as well as fair distribution of power and recognition. Making a plan and clearly communicating expectations for all contributors to ensure both of these aspects are equitable, or at least commensurate with one another, can promote fairness [102]. Norms around what constitutes “enough” of a contribution to become an author differ by field. Similarly norms about the relationship between author order and the prestige or community recognition of the work, either explicitly or implicitly, vary greatly by field [103]. It is important to have conversations early on in the process about expected contributions and authorship to make sure there are no mismatches in expectations and outcomes.

A study of author contribution statements in the journal *PLOS ONE* revealed different patterns in the division of labor in research teams [104]. One of the team’s findings showed that interdisciplinary projects tend to have less division of labor; instead, every part of the team works on multiple parts of the process. The authors propose one possible explanation for this: members “integrate these different perspectives by collaborating more closely on the same activities.” Data-intensive research teams often bring together people from a variety of backgrounds and with heterogeneous expertise. To avoid teammates being “siloed” into only the roles for which they have the comparative advantage, including multiple people in each step of the workflow can both ensure multiple perspectives are weighing in on each step but also give team members an opportunity to expand their skill sets.

Logistics constraints can make responsible collaboration challenging as teams understandably are pressured to do more with less (time and/or money). For example, Eitzel [105] documents how a member of an interdisciplinary working group decided to stop attending group meetings because of a mix of logistical burdens (heavy teaching load and a conference deadline) and personal burdens (always being called upon to teach the group about the basics of their discipline while simultaneously having the discipline being undervalued by the group members from another discipline). Eitzel [105] perceived this group member’s missing contributions to the discussions after leaving to be a huge loss for the group’s overall understanding and progress. Valuing the group member’s expertise both in attitude and compensation would have been helpful in maintaining their contributions to the overall group.

Team diversity, decentralization, and inclusion are important for ethical as well as epistemic reasons. Research findings from decentralized and non-repeat collaborations were found to be more likely to be replicated in subsequent research as they have been tested across different methods and conditions [106]. Gender-diverse teams have been found to generate more novel and creative ideas [107], particularly when diversity is considered together with inclusion [108]. Yet, women are underrepresented in fields related to data-intensive research, including computer science and software development [109], and are more likely to leave compared to men [110].

## Rule 8: Prioritize continual learning and reflection to embed ethical considerations throughout the entire research process rather than just at the beginning and end

Responsible workflows require an iterative approach and constant questioning about consequences and discussion of all relevant stakeholders, not just the obvious ones. However, a team decides to implement continual learning and reflection, conversations about ethics should be ongoing throughout a project. Zook and colleagues also highlight responsibility in data work. In their rules for responsible big data research, they identify ethical action items throughout a research project, highlighting the constant responsibility necessary in data work [111].

For an example early on in the research process, the Stanford Institute for Human-Centered Artificial Intelligence piloted an “ethics and society review board” that required consideration of risks towards broader society in addition to the human subject that is more typical of the IRB process for health-related research, in order to apply to grant funding. In addition, this review process also included iterative feedback for applicants to further reflect and revise their plans [112].

Finding a framework for prompting reflection that works for a team can be challenging. Eitzel [105,113] presents one such framework for autoethnographic assessment of best practices. Eitzel [105,113] provides an example of working through the process herself on her own modeling best practices that includes social science considerations as well as more traditional modeling practices. This process helped Eitzel not only grapple with issues of technical reproducibility (by transparently providing errata to a previously published paper when a mistake was found in the analysis) but also helped improve her modeling practices by working to engage with community stakeholders. This occurred by interviewing researchers at a field station about oak tree data collection procedures and creating a data biography that helped when synthesizing multiple datasets into one conservation analysis.

## Rule 9: Make sure your responsible data science workflow is sustainable. This includes aligning incentives and giving people credit for responsible research

Once you build your responsible data science workflow, it becomes a research object on its own and it needs to be maintained and updated like other FAIR research objects [114]. Responsible data science workflows are composite workflows consisting of heterogeneous research objects, computational infrastructure, and socio-technical tools. Many of these objects are not typically considered as part of a workflow (for example, data ethics, team formation, authorship allocation). Once your workflow involves heterogeneous processes, you need to keep these processes together by documenting them. You can use GitHub, Open Science Framework, or another repository to document your workflow and link the various objects from team formation, through research design and data analysis, to dissemination of results.

Compared to automated computational workflows, which are precise descriptions of procedures [114–116], a responsible data science workflow is a broader notion, incorporating principles of fairness and equity applied to data subjects, research collaborations, and model outputs. Consequently, in comparison to automated computational workflows, many processes of responsible workflows may be less crystalised in current data science research and may also be less codifiable, recordable, accessible, interoperable, and reusable. Nevertheless, some processes are in the process of getting codified.

For example, fair recognition of individual contributions to a research output can be very difficult in interdisciplinary data science projects, especially for Early Career Researchers (ECR), yet tools such as the CRediT (Contributor Roles Taxonomy) taxonomy allow authors to provide a precise description of their contributions to the published work, enabling a fair allocation of research credit. Relatedly, one of the authors (BM) outlines principles of Authorship Ethics on their personal website, synthesizing from a variety of resources [117–119].

The maintenance of a responsible data science workflow requires effort and resources. However, incentives in the current reward system of scientific research and of competitive industries are often misaligned with responsible data science and ML applications, favoring novel positive findings at the expense of model transparency, reproducibility, or bias evaluation [21,23,24,120]. Performance assessment of researchers for hiring, promotion, and tenure often considers metrics such as the number of published papers, citations, and Journal Impact Factor (JIF) [121]. When extra time is put into the quality of the work, i.e., making work ethical and reproducible, this can come at the expense of quantity. This reality should be accounted for in performance reviews of people who do this work, such that responsible data-intensive research is rewarded instead of penalized. In industry settings, incentives can be different and may include recognition for unsung work in the form of promotion and monetary raises. However, the same tension between quality and quantity remains and should be considered when making decisions about a researcher’s progress. Recently, the realization of misaligned incentives also led to new research reforms, scientific communities, and regulations aiming at responsible data-intensive research. Many journals and conferences encourage and promote the availability of code and data. Further, in 2023, the National Institutes of Health (NIH) introduced a Data Management and Sharing Policy that mandates the sharing of scientific data. As regulations and institutional reforms are only in early stages, incentives for doing ethical and reproducible work come mostly from the research communities. Therefore, it is important to give people credit for following the rules of a responsible workflow when reviewing their work in our own communities and spheres of influence.

## Rule 10: Communicate your workflow along with your research outputs to a wide audience, including the communities you are responsible to

Part of the maintenance of a project includes documenting the entire process. Sharing that process frequently, to stakeholders and colleagues alike, rather than only through a written document or oral presentation at the end of the project, can help others learn about both your findings and your workflow. Some may benefit more from hearing about the team’s workflow itself so that they can repurpose the approach for another research aim. Being transparent about the behind-the-scenes work, from what worked to what did not, can help others streamline their workflows and avoid the same pain points and build trust within the communities affected by the work.

Communicating about the work throughout the process rather than just at the end can also benefit the research team by keeping them accountable for keeping materials organized and transparent [122]. This communication does not always have to occur in formal venues. For example, various data-related organizations have a blog where members of the group explain the processes behind their work for a wider audience (e.g., [123–125]). Other venues celebrate the process behind the work rather than only the outputs themselves (e.g., [126,127]) or give researchers a chance to make their formal work more accessible to a general audience (e.g., [128,129]). Valuing nontraditional communication venues and mediums within a team can encourage creative contributions that showcase the work of the team while also helping to reach communities who might not otherwise engage with the work. These informal mediums can help make the work more accessible, increasing its impact, and bring more people into the conversation about the choices made in the project.

## Conclusions

A responsible data science workflow combines technical and social considerations throughout the whole lifecycle of a project. By recognizing that we as researchers are responsible to many different stakeholders, with potentially competing interests and who are differentially impacted by the work, in an ever-changing context and data environment, we can make better choices about the technical side of the project. We need to act ethically and be adaptable to ensure we remain ethical as new information arises.

Our goal with these 10 rules is not to provide a whole new workflow to switch to but rather to combine much of the “best practices wisdom” into one workflow that also acknowledges the human component of both the work and the impact of data-intensive research.
